# ‘EarlyAMDRate’: A grading instrument for OCT‐based assessment of early lesions caused by age‐related macular degeneration

**DOI:** 10.1111/aos.17479

**Published:** 2025-03-30

**Authors:** Marcus Wagner, Thomas Peschel, Carla J. Leutloff, Franziska G. Rauscher

**Affiliations:** ^1^ Institute for Medical Informatics, Statistics and Epidemiology (IMISE) Leipzig University Leipzig Germany; ^2^ Department of Ophthalmology Martin Luther University Halle‐Wittenberg Halle Germany; ^3^ Department of Medical Data Science, Medical Informatics Center Leipzig University Medical Center Leipzig Germany; ^4^ Experimental and Clinical Research Center, Max Delbrück Center for Molecular Medicine Berlin Germany; ^5^ Neuroscience Clinical Research Center, Charité—Universitätsmedizin Berlin, Corporate Member of Freie Universität Berlin and Humboldt‐Universität zu Berlin Berlin Germany; ^6^ Berliner Hochschule für Technik (BHT) Berlin Germany; ^7^ Leipzig Research Centre for Civilization Diseases (LIFE) Leipzig University Leipzig Germany

**Keywords:** age‐related macular degeneration, early lesions, grading questionnaire, manual grading, metric phenotypes, optical coherence tomography

## Abstract

**Background and Objectives:**

Long before any signs of age‐related macular degeneration (AMD) become clinically noticeable, the disease starts with accumulation of deposits of extracellular debris and formation of lesions within the outermost layers of the retina. For a reliable imaging of lesions in these early stages, optical coherence tomography (OCT) turned out to be largely preferable to colour fundus photography. However, an adequate grading instrument for Early‐AMD lesions within OCT data is missing in the literature as yet. The present paper aims to fill this gap.

**Methods:**

‘EarlyAMDRate’, an instrument for OCT‐based grading of Early‐AMD lesions, is presented and documented. It comprises a questionnaire assessing a given lesion with respect to its relative position and interaction with the surrounding retinal layers, its brightness, special properties and state of progression (if applicable). Furthermore, the grading procedure includes a graphical masking of the lesion within the OCT image.

**Results:**

For a consecutive sample of *N* = 100 Early‐AMD patients, the ‘EarlyAMDRate’ grading instrument has been applied to leading OCT scans. Examples of masked lesions and processed grading questionnaires are provided. Both raw lesion diameters and cutting sizes follow a log‐normal sample distribution.

**Conclusions:**

‘EarlyAMDRate’ allows for unprecedented detail of description for single Early‐AMD lesions which is adequate to the precision of underlying OCT imaging. The obtained grading information allows for a tracking of single lesions and their properties over time as well as for the generation of well‐differentiated metric phenotypes for description of Early‐AMD.

## 1 INTRODUCTION

### 1.1 Scientific background

Age‐related macular degeneration (AMD), a progressive retinal disease without a known cure, is the leading cause of blindness in the elderly; see Chakravarthy and Peto (2020), Finger et al. (2011) and Li et al. (2020). Age and smoking, cf. Cheung and Wong (2014), as well as a strong, multifactorial genetic predisposition, cf. DeAngelis et al. (2017) and Winkler et al. (2020), constitute the main risk factors. Current knowledge is largely confined to late AMD with clinically evident visual impairment. However, long before any signs of AMD become clinically noticeable, the disease starts with accumulation of deposits of extracellular debris within the outermost layers of the retina, evolving into different types of lesions such as drusen or subretinal drusenoid deposits (SDDs), cf. Spaide et al. (2018). Very little is known about prevalence, dynamics and reversibility of these early lesions and their driving forces.

For the investigation of these early stages of the disease, fundus‐image‐based phenotyping turned out to be largely inadequate since (a) Early‐AMD lesions may remain completely invisible in fundus images or be not clearly identifiable therein, cf. Pead et al. (2019), and (b) a reliable discrimination of different lesion types (e.g., hard drusen vs. SDDs) in the fundus is impossible, cf. Spaide et al. (2018). Consequently, for a closer investigation of Early‐AMD lesions, imaging techniques beyond fundus photography must be employed. Retinal optical coherence tomography (OCT), cf. Huang et al. (1991), is particularly suitable here because (a) early stages of lesions invisible in fundus images will be reliably captured by OCT, thus made gradable and measurable, while the lesions noticeable in fundus imaging can be unambiguously re‐identified within OCT scans, and (b) in most cases, different lesion types can be mutually distinguished by their spatial location, shape and reflectivity pattern, cf. Spaide et al. (2018), Sect. 6.2, and Zweifel et al. (2010). In particular, hard drusen and SDDs can be differentiated within OCT images by their position relative to the outermost RPE layer, cf. Spaide et al. (2018), p. 784, Figure 1 and Gattoussi et al. (2019), p. 367, Figure 2.

However, the precision and detail achieved in OCT‐based imaging of Early‐AMD lesions sharply contrasts with the traditional coarseness of its description and annotation. While for the retinal layers and reflectivity bands visible in OCT, an adequately detailed, finely structured nomenclature is available, cf. Staurenghi et al. (2014), pathological retinal features as lesions, macular holes, hyper‐reflective foci etc. have been only superficially and phenomenologically classified as yet, cf. Gattoussi et al. (2019). Likewise, none of the generally accepted AMD classification schemes, cf. Bird et al. (1995), Davis et al. (2005), Ferris et al. (2013), Klaver et al. (2001); Klein et al. (1991), Klein et al. (2014) and Korb et al. (2014), all of them fundus‐based and categorial, makes use of the detailed information captured in OCT data. In fact, cutpoints and thresholds within these classifications are still defined by cumulative lesion properties, for example, total number of observed lesions, total area occupied by lesions or maximal diameter among all observed lesions. An adequate grading instrument, allowing for sufficiently detailed annotation and description of single Early‐AMD lesions within OCT data as well as for generation of metric phenotypes for AMD stage evaluation is missing as yet. The present paper is intended to fill this gap.

### 1.2 Aims of the paper

We introduce and describe ‘EarlyAMDRate’ as a grading instrument for OCT‐based description of single Early‐AMD lesions. ‘EarlyAMDRate’ comprises a questionnaire assessing a given lesion with respect to its relative position and interaction with the surrounding retinal layers, its brightness, special properties and state of progression (if applicable). Additionally, the grading procedure includes a graphical masking of the lesion within the OCT image.

The main purposes of the ‘EarlyAMDRate’ instrument are fourfold.

1. The instrument provides an *OCT‐based standard for* the *definition of Early‐AMD lesion properties*, providing sufficiently detailed categories for its description.
2. The instrument is suitable for *OCT‐based manual grading of single Early‐AMD lesions*, enabling a reliable identification of lesions in its early and even earliest stages, thus making detailed ground‐truth information available.
3. Lesion masking by ‘EarlyAMDRate’ allows for the *generation of metric phenotypes* for individual lesions as well as for whole OCT data sets.
4. *Tracking of individual lesions and assessment of lesions' progression* throughout several examinations will be made possible.

The ‘EarlyAMDRate’ instrument is *not* an automated procedure. However, it is well suitable for embedding into a larger framework of OCT data analysis. Assessment categories of this scheme may be easily incorporated into future platforms for automated recognition, description and classification of lesions.

The present study is focused on the introduction and description of the grading instrument, including a typical example for its practical use. Namely, a consecutive sample of *N* = 100 Early‐AMD patients will be assessed with respect to the lesions visible in the leading OCT scans. To the contrary, details of the realization and software environment will not be covered by this paper.

### 1.3 Outline

In Section 2, we describe first the ‘EarlyAMDRate’ grading instrument in full detail. Further, we document the selection of a consecutive sample of Early‐AMD patients from the LIFE‐Adult cohort, the details of OCT imaging applied and grading, as well as the plan for analysis of the grading results. In Section 3, the results are presented. The paper continues with a discussion (Section 4) and a conclusion (Section 5). A detailed working instruction for the usage of the ‘EarlyAMDRate’ grading instrument is provided in Appendix A.

## 2 MATERIALS AND METHODS

### 2.1 ‘EarlyAMDRate’, a grading instrument for precise description of single Early‐AMD lesions

**FIGURE 1:**
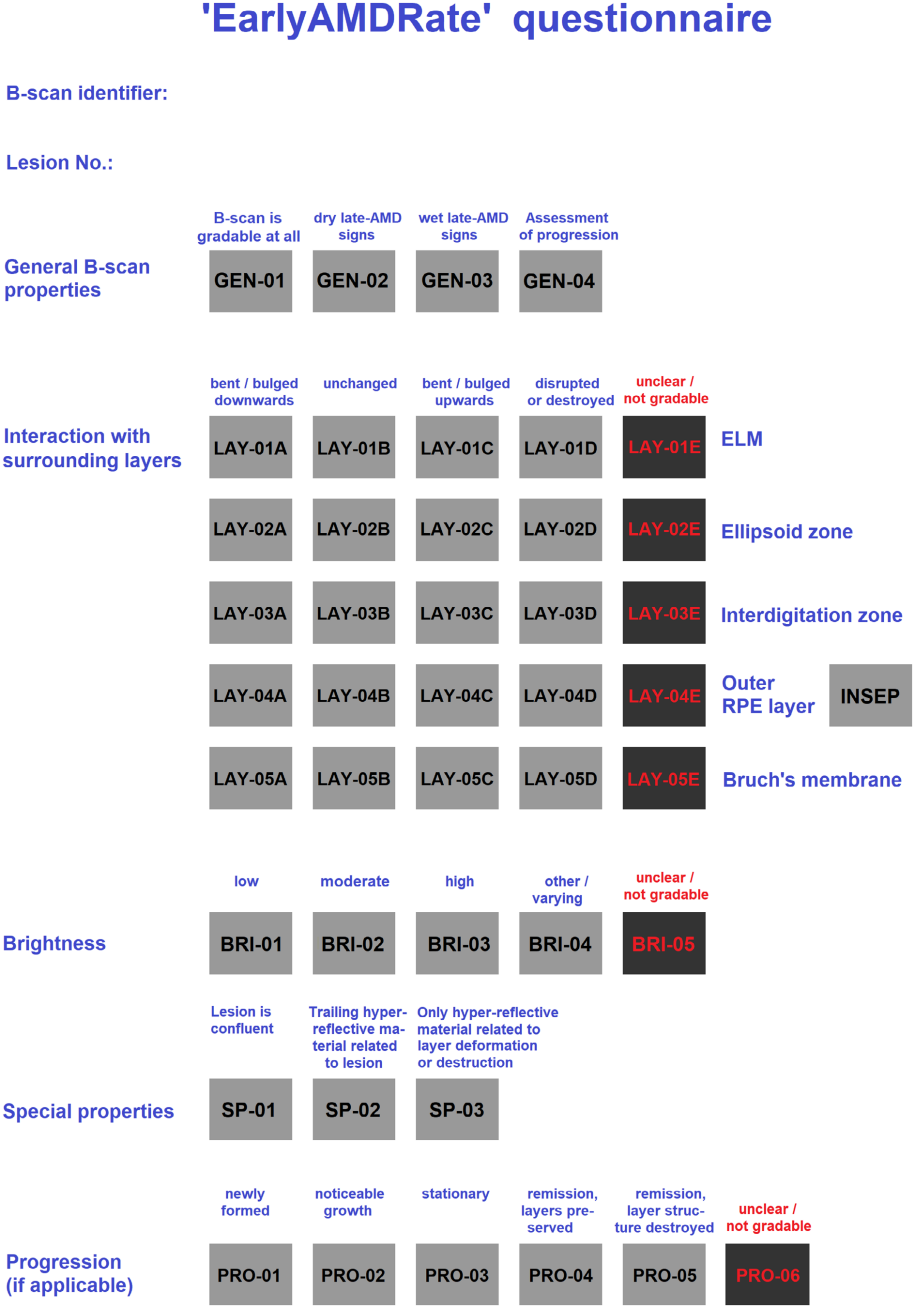
The ‘EarlyAMDRate’” questionnaire. Fields must be ticked according to the definitions in Section 2.1.1 and the working instruction in Appendix A.

The grading instrument ‘EarlyAMDRate’ is intended for the description of single Early‐AMD lesions within OCT scans. Lesion properties will be assessed by processing a questionnaire, cf. Figure 1, and lesion shape and size will be captured and measured by graphical masking of the lesion within the scan.

#### 2.1.1 Components of the questionnaire

The ‘EarlyAMDRate’ questionnaire is always related to a single lesion. Nevertheless, some general properties of the OCT scan under consideration are recorded (fields GEN‐01 to GEN‐04). Namely, the questionnaire asks whether the scan is gradable at all, whether signs of dry or wet Late‐AMD are visible in the scan, and whether the scan under consideration is a follow‐up image, which is assessed together with its related baseline scan.

Now the questionnaire turns to the description of the particular lesion, asking first for a detailed assessment of the surrounding retinal layer structure (fields LAY‐01A to LAY‐05E). The following layers/reflectivity bands are taken into account: external limiting membrane (ELM), ellipsoid zone (EZ), interdigitation zone (IZ), outer retinal pigment epithelium complex (RPE) and Bruch's membrane (BM). The definitions of these features follow Staurenghi et al. (2014) and Wagner et al. (2025a). For every layer, we assess whether (1) it is bent or bulged downwards but still connected, or (2) unchanged and intact, or (3) bent or bulged upwards but still connected, or (4) disrupted or destroyed or (5) its state and geometry cannot be clearly assessed, thus being not gradable. Further, it must be indicated whether interdigitation zone and outer RPE layer can be clearly separated around the lesion or not (field INSEP). In most cases, the relative position of a given lesion can be unambiguously derived from this information by checking the layers whose affection by the lesion is rated by (1) or (2).

Second, the brightness of the lesion is assessed (fields BRI‐01 to BRI‐05). Possible descriptions are (1) low brightness, (2) moderate brightness, (3) high brightness, (4) other (e.g. brightness strongly varies throughout the lesion) or (5) unclear or not gradable at all.

Third, the questionnaire asks for three special properties (fields SP‐01 to SP‐03), namely whether (1) the lesion is confluent, (2) in relation to the lesion, hyper‐reflective material trailing into the inner retina (‘drusen ooze’) is present, cf. Monés et al. (2017) and (3) the lesion consists of hyper‐reflective material only, which is related to layer deformation or destruction.

Fourth, in the case of simultaneous treatment of baseline and follow‐up scans at identical positions, the state of progression of the lesion at follow‐up is assessed, see Section 2.1.3 below.

A detailed working instruction for processing the ‘EarlyAMDRate’ questionnaire and performing the lesion masking is provided in Appendix A. Manual grading of lesions should be performed by persons with ophthalmological expertise.

#### 2.1.2 Masking of lesions

Masking assumes that the B‐scan under consideration is available as a writable greyscale image. Lesion masks should be drawn in (using colour instead of white, black or greyscale tones). Every lesion mask must form a connected area (horizontal, vertical or diagonal connections between pixels are possible). The minimal size of a mask is 2 pixels. If more than one lesion is present in a given B‐scan, every lesion should be masked with a different colour.

From the lesion masks, geometrical properties of lesions such as masked area, projected diameter and coordinates of its centre can be derived, measured in pixel units. Usually, OCT devices provide absolute dimensions of pixels in micrometres calculated by internal, mostly undocumented software. If, however, a measurement of the corneal radius is available, then the pixel width can be explicitly calculated from this value and the scan focus by the Garway‐Heath formula, cf. Garway‐Heath et al. (1998), p. 649.

#### 2.1.3 Tracking of lesions

Assume that a pair of related scans at baseline and follow‐up at identical positions is available. Then, for every lesion in the follow‐up scan, the state of progression can be assessed (fields PRO‐01 to PRO‐06). The possibilities are that the lesion (1) is newly formed, (2) shows noticeable growth, (3) is stationary, (4) shows a partial or complete remission while the surrounding layer structure is preserved, (5) shows a partial or complete remission but the surrounding layer structure is destroyed or (6) the progression state is unclear or cannot be assessed at all. Clearly, cases (2) to (5) require an unambiguous identification of the follow‐up lesion under consideration with a corresponding lesion image in the baseline scan.

#### 2.1.4 Remarks about software environment

Currently, the “EarlyAMDRate” grading instrument is embedded into a set of MATLAB procedures. A given B‐scan will be converted into a larger grading form. After manual processing, an automated readout and storage of grading information are realized. All procedures were tested on MATLAB 9.14.0.2286388 (R2023a), requiring the MATLAB Image Processing Toolbox (documented at mathworks.com/products/matlab and mathworks.com/products/image, both accessed 11 January 2025). No particular attempts at optimization of runtime behaviour were made. This software is made accessible under licence CC BY‐SA 4.0 (creativecommons.org/licenses/by‐sa/4.0, accessed 11 January 2025) at the Leipzig Health Atlas repository, cf. Kirsten et al. (2022), and can be reached from the website health‐atlas.de/data_files/616.

### 2.2 Application of the ‘EarlyAMDRate’ instrument to a consecutive sample of Early‐AMD patients

#### 2.2.1 The LIFE‐Adult cohort

The Leipzig Research Center for Civilization Diseases (LIFE)‐Adult study is a population‐based cohort study, for which 10000 randomly selected inhabitants of the city of Leipzig (Saxonia, Germany) between 40 and 79 years of age were recruited and deeply phenotyped between 2011 and 2014, cf. Loeffler et al. (2015) and Engel et al. (2023). During the baseline and 6‐years follow‐up examinations, ophthalmological imaging data were generated for more than 9000 and 1800 adults, respectively. For the current research, we employed OCT volume scans of the macula region. The LIFE Adult study follows the tenets of the Declaration of Helsinki and was approved by the responsible institutional ethics board of the Medical Faculty of Leipzig University (approval numbers 2632009‐14122009, 263/09‐ff, 201/17‐ek). Written informed consent was obtained from all participants. Data use was approved by the institutional review board of the Leipzig Research Center for Civilization Diseases (LIFE).

#### 2.2.2 Selection of the Early‐AMD sample

For the LIFE‐Adult Follow‐up cohort, the last author performed OCT‐based AMD grading for both eyes, using the Three‐Continent Classification (TCC) scheme from Klein et al. (2014). This scheme defines five severity steps of AMD (no, mild early, moderate early, severe early and late). Early‐AMD in the sense of our work comprises the mild early, moderate early and severe early severity categories. Additionally, eyes classified by TCC as No‐AMD but with apparent lesions in OCT imaging are counted as Early‐AMD as well. Consequently, No‐AMD eyes in our study satisfy the TCC condition of No‐AMD, which is strengthened in the sense that lesions are completely absent. In the following, a person is classified as Early‐AMD if either both eyes are Early‐AMD or one eye is Early‐AMD and the other one is No‐AMD.

From all 302 consecutive patients undergoing the follow‐up visit from 1 November 2019 to 9 June 2020, we selected all persons classified as Early‐AMD by the rules described above and born in 1949 or before, thus aged 70 years or more at the date of the visit. Thus, we arrived at a study population of 100 patients. If both eyes of a study patient were classified as Early‐AMD, then one eye was randomly selected by rolling a dice. Otherwise, the single eye classified as Early‐AMD has been chosen. The characteristics of the study population are summarized in Table 1.

**TABLE 1:** Properties of the Early‐AMD study population.

Entity	Male	Female	Total
Sex	52	48	100
Age (years)	77.5 ± 3.8	76.3 ± 4.1	76.9 ± 4.0
BMI (kg/m^2^)	27.69 ± 3.54	27.37 ± 4.74	27.54 ± 4.13
Early‐AMD eyes available (OD/OS/Both)	13/10/29	8/7/33	21/17/62
Assigned from both by dice (OD/OS)	17/12	14/19	31/31
Selected Early‐AMD eye (OD/OS)	30/22	22/26	52/48

#### 2.2.3 OCT image acquisition

Optical coherence tomography data were generated by a commercially available spectral‐domain device (Spectralis HRA + OCT, equipped with camera head with serial number 04514, software modules Heidelberg Eye Explorer 1.7.0.0, Acquisition Module 5.4.7.0 and Viewing Module 5.4.6.0; Heidelberg Engineering (heidelbergengineering.com, accessed 13.06.2024). This device works with a super luminescent diode at a central wavelength of 870 nm. For every participant, volume scans of the macular area with a field size of 20° (temporal–nasal) × 20° (superior–inferior) were acquired for both eyes. Each volume scan consists of 97 equally spaced B‐scans of 496 × 512 pixel size. Scan depth in tissue is about 1.9 mm, resulting in a pixel depth of 3.87 μm while the axial optical resolution amounts to 7 μm, cf. Heidelberg Engineering GmbH (2011), p. 273. The real‐time eye‐tracking function of the device was enabled, thus obtaining an average of 10 measurements per column. No use of special imaging modules was made. Raw data were exported and used, cf. Heidelberg Engineering GmbH (2008), and subsequently scaled with the fourth root and greyscale‐binned, thus being converted into classical visualization as a greyscale image, see Wagner et al. (2025a), Sect. 2.3.1., *gimdat* mode. Pixel width was calculated by the internal software of the OCT device and reported during the raw data export.

#### 2.2.4 Identification of leading drusen and leading scan

Within every volume scan, the leading B‐scan and the leading drusen were identified by visual inspection. The leading drusen is defined as the lesion with the maximal cutting area visible in a B‐scan throughout the volume, and the leading scan is defined as the B‐scan showing the leading drusen. This procedure resulted in the selection of *N* = 100 leading scans containing a total of *N* = 198 lesions.

#### 2.2.5 Manual grading of Early‐AMD lesions

Within every leading scan, all visible lesions were manually graded by use of the ‘EarlyAMDRate’ instrument. In particular, all lesions were graphically masked, thus obtaining the size of their cutting area and their projected diameter. Progression state was not assessed.

### 2.3 Presentation and analysis of grading results

#### 2.3.1 Descriptive statistics of lesion properties

For the fields of the ‘EarlyAMDRate’ questionnaire, we report the occupancies observed in grading. Moreover, we classify the lesions by their special properties and by the state of Bruch's membrane and outer RPE layer into drusen, SDDs and hyper‐reflective foci. Progression state was not assessed.

#### 2.3.2 Selection of grading examples

Selected examples comprise hard drusen of different brightness, SDDs and hyper‐reflective foci. Original images, lesion masks and processed questionnaires will be provided. Cutouts pictured have 300 × 300 pixels format. Insets show lesions or lesion masks in double magnification. In all examples, the progression state was not assessed.

#### 2.3.3 An additional example of lesion tracking

In order to illustrate lesion tracking, for a single Early‐AMD proband from the sample, the leading scan from follow‐up was examined together with the related baseline scan. Original images, lesion masks and gradings including progression assessment will be reported. Cutouts pictured have 300 × 300 pixels format.

#### 2.3.4 Correlation between measurement units

For every lesion, the projected diameter and size of the cutting area will be obtained from its mask, both measured in pixels. Using the built‐in software of the OCT device, these values will be converted into μm or μm^2^ units. Pearson's correlation coefficients between measurements in pixels and μm or μm^2^ units will be calculated.

#### 2.3.5 Distribution of raw lesion sizes

Observed raw values of projected lesion diameters and lesions' cutting area sizes, as converted in μm or μm^2^ units, will be described by sample statistics and plotted into histograms. Log‐normality of value distributions will be assessed by the Shapiro–Wilk test, cf. Shapiro and Wilk (1965).

## 3 RESULTS

### 3.1 Descriptive statistics of lesion properties

**FIGURE 2:**
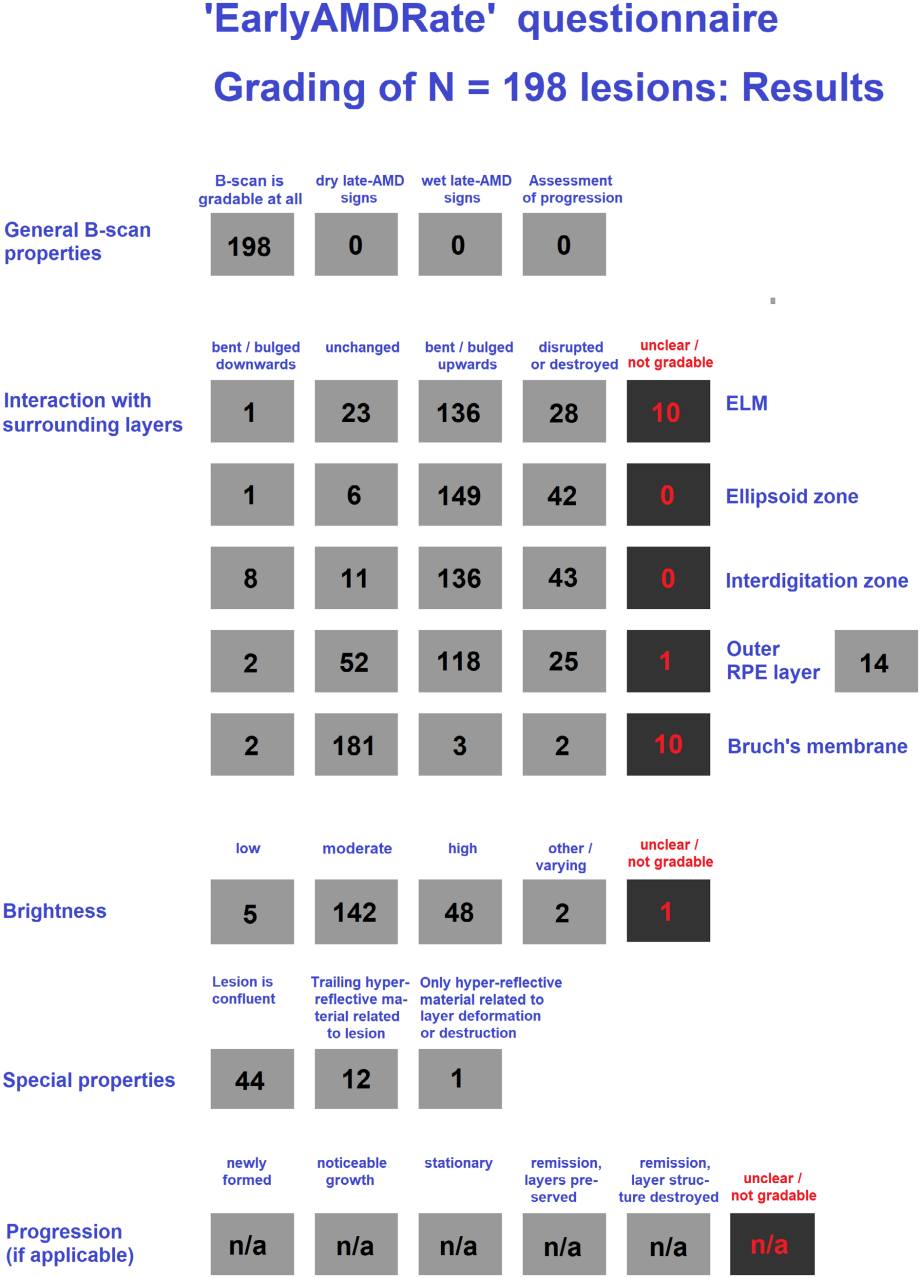
Grading results for *N* = 198 Early‐AMD lesions from the study population. Progression state has not been assessed. Fields of the questionnaire are defined in Figure 1 above.

In Figure 2, the results of manual grading are summarized. The sample comprises 100 B‐scans, all of them gradable and without any signs of Late‐AMD, containing a total of *N* = 198 lesions. Of these, 1 lesion consists of hyper‐reflective material only (field SP‐03 ticked), 54 lesions bent the outer RPE layer down or leave it unaffected (fields LAY‐04A or LAY‐04B ticked), thus being operationally defined as SDDs, 142 lesions bent the outer RPE layer upwards or break through (fields LAY‐04C or LAY‐04D ticked), thus being considered as drusen, and 1 remaining lesion lacks information about the RPE state. Forty‐four lesions are confluent.

### 3.2 Examples for single lesion gradings

**FIGURE 3:**
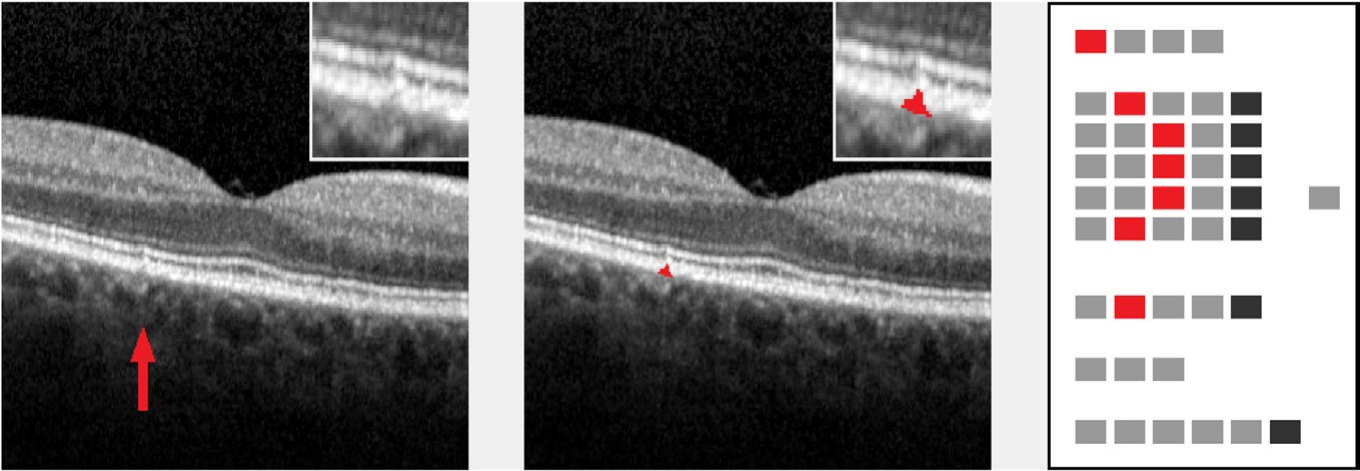
Small drusen with moderate brightness (proband #32). Left: original image (cutout), lesion marked by arrow. Inset: lesion in double magnification. Middle: lesion mask (cutout), raw cutting area is 1705 μm^2^. Inset: lesion mask in double magnification. Right: processed ‘EarlyAMDRate’ questionnaire. Marked fields are GEN‐01 (B‐scan is gradable at all), LAY‐01B (ELM is unchanged), LAY‐02C, LAY‐03C, LAY‐04C (EZ, IZ and outer RPE layer are bent/bulged upwards), LAY‐05B (BM is unchanged) and BRI‐02 (moderate brightness), see Figure 1.

**FIGURE 4:**
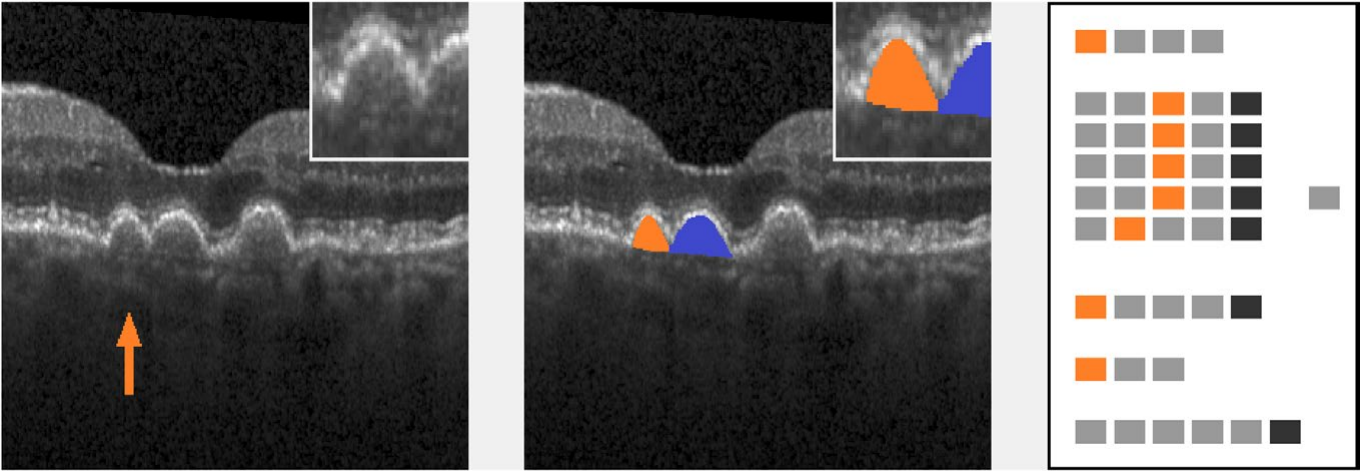
Confluent drusen with low brightness (proband #40). Left: original image (cutout), lesion marked by arrow. Inset: lesion in double magnification. Middle: lesion mask (cutout), raw cutting area is 15 228 μm^2^. Inset: lesion mask in double magnification. Neighbouring lesion is masked in blue to show vertical delineation between confluent lesions. Right: processed ‘EarlyAMDRate’ questionnaire. Marked fields are GEN‐01 (B‐scan is gradable at all), LAY‐01C, LAY‐02C, LAY‐03C, LAY‐04C (ELM, EZ, IZ and outer RPE layer are bent/bulged upwards), LAY‐05B (BM is unchanged), BRI‐01 (low brightness) and SP‐01 (lesion is confluent), see Figure 1.

**FIGURE 5:**
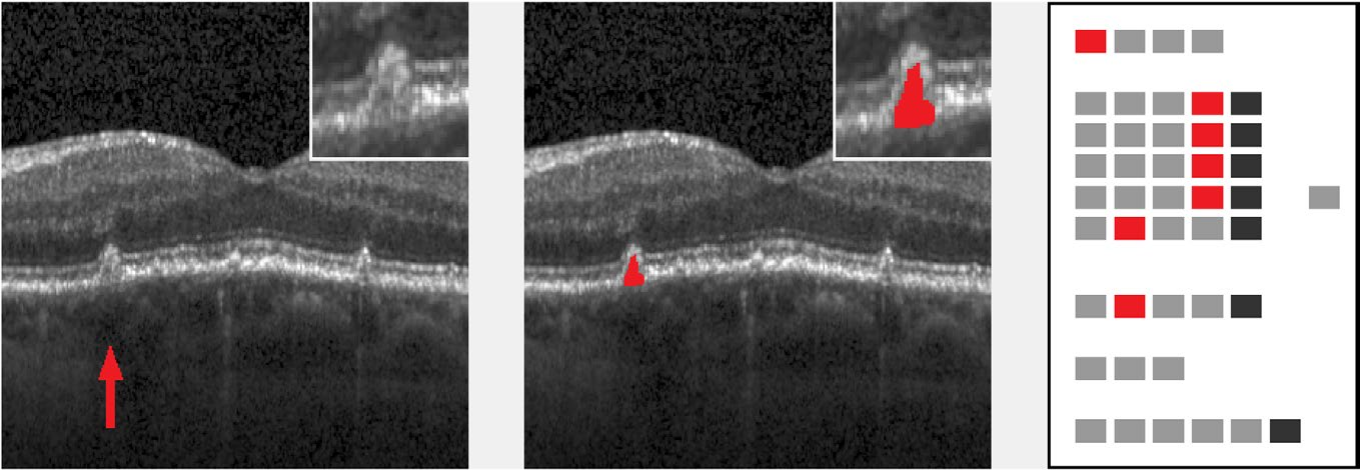
Drusen, disrupting the outer retinal layers (proband #43). Left: original image (cutout), lesion marked by arrow. Inset: lesion in double magnification. Middle: lesion mask (cutout), raw cutting area is 7645 μm^2^. Inset: lesion mask in double magnification. Right: processed ‘EarlyAMDRate’ questionnaire. Marked fields are GEN‐01 (B‐scan is gradable at all), LAY‐01D, LAY‐02D, LAY‐03D, LAY‐04D (ELM, EZ, IZ and outer RPE layer are disrupted/destroyed), LAY‐05B (BM is unchanged) and BRI‐02 (moderate brightness), see Figure 1.

**FIGURE 6:**
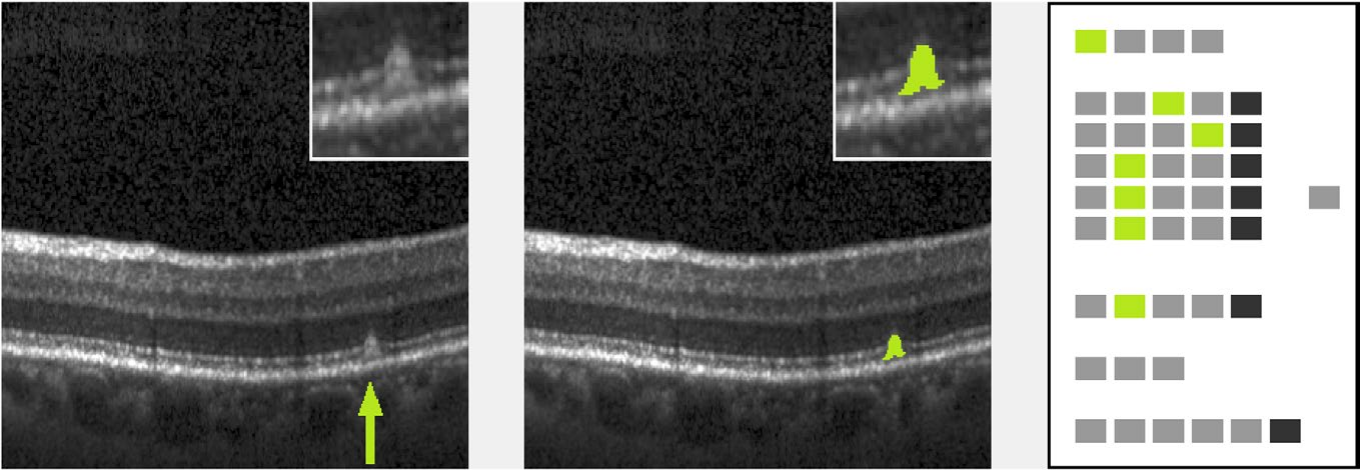
SDD (proband #20). Left: original image (cutout), lesion marked by arrow. Inset: lesion in double magnification. Middle: lesion mask (cutout), raw cutting area is 5292 μm^2^. Inset: lesion mask in double magnification. Right: processed ‘EarlyAMDRate’ questionnaire. Marked fields are GEN‐01 (B‐scan is gradable at all), LAY‐01C (ELM is bent/bulged upwards), LAY‐02D (EZ is disrupted/destroyed), LAY‐03B, LAY‐04B, LAY‐05B (IZ, outer RPE layer and BM are unchanged) and BRI‐02 (moderate brightness), see Figure 1.

**FIGURE 7:**
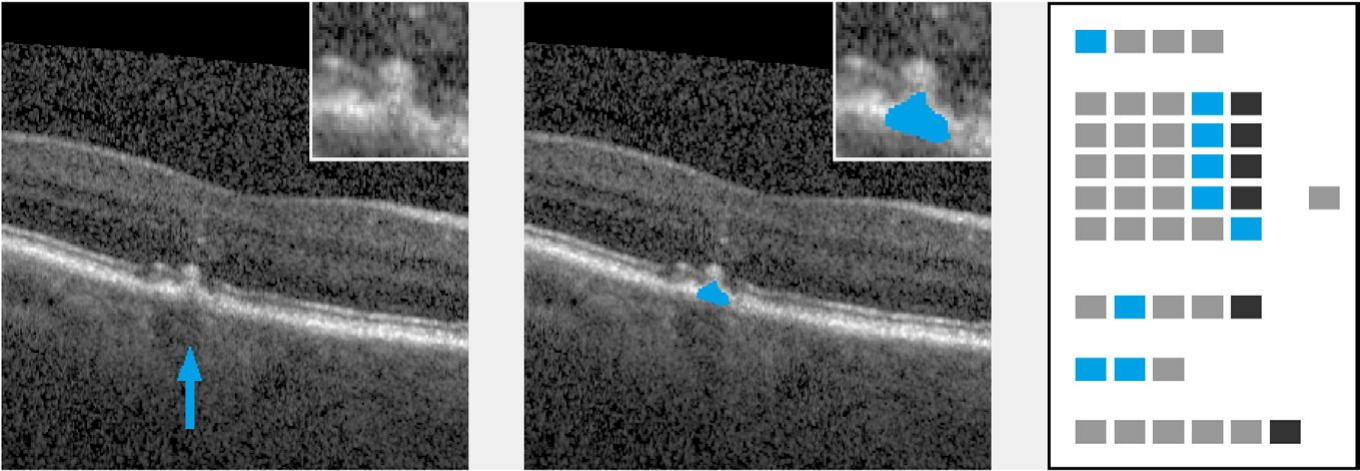
Lesion with related hyper‐reflective material trailing into inner retina (proband #83). Left: original image (cutout), lesion marked by arrow. Inset: lesion in double magnification. Low image quality allows only for partial assessment of the lesion. Middle: lesion mask (cutout), raw cutting area is 8362 μm^2^. Inset: lesion mask in double magnification. Right: processed ‘EarlyAMDRate’ questionnaire. Marked fields are GEN‐01 (B‐scan is gradable at all), LAY‐01D, LAY‐02D, LAY‐03D, LAY‐04D (ELM, EZ, IZ and outer RPE layer are disrupted/destroyed), LAY‐05E (BM is not gradable), BRI‐02 (moderate brightness), SP‐01 (lesion is confluent) and SP‐02 (trailing hyper‐reflective material related to lesion is present), see Figure 1.

**FIGURE 8:**
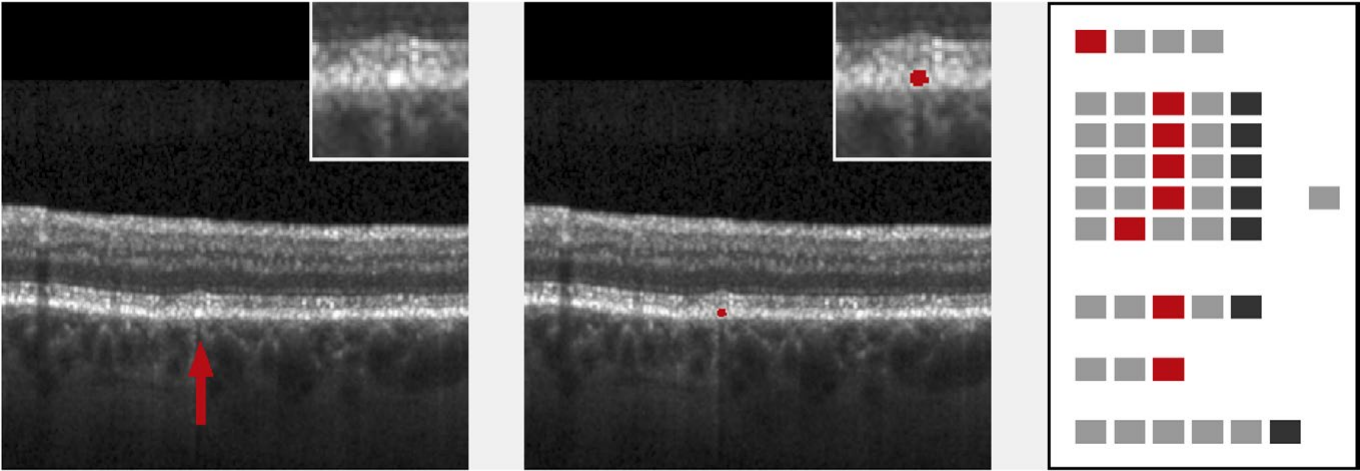
Lesion consisting of hyper‐reflective material only (proband #10). Left: original image (cutout), lesion marked by arrow. Inset: lesion in double magnification. Note that hyper‐reflective material shadows below. Middle: lesion mask (cutout), raw cutting area is 2362 μm^2^. Inset: lesion mask in double magnification. Right: processed ‘EarlyAMDRate’ questionnaire. Marked fields are GEN‐01 (B‐scan is gradable at all), LAY‐01C, LAY‐02C, LAY‐03C, LAY‐04C (ELM, EZ, IZ and outer RPE layer are bent/bulged upwards), LAY‐05B (BM is unchanged), BRI‐03 (high brightness) and SP‐03 (lesion consists of hyper‐reflective material only which is related to layer deformation), see Figure 1.

In Figures 3, 4, 5, 6, 7, 8, selected examples of single lesions' gradings are presented. Examples comprise drusen of different shapes and brightness, a SDD, a hyper‐reflective focus and a lesion with trailing hyper‐reflective material. Cutouts of 300 × 300 pixels format are shown together with processed ‘EarlyAMDRate’ grading questionnaires.

### 3.3 Example for tracking of lesions

**FIGURE 9:**
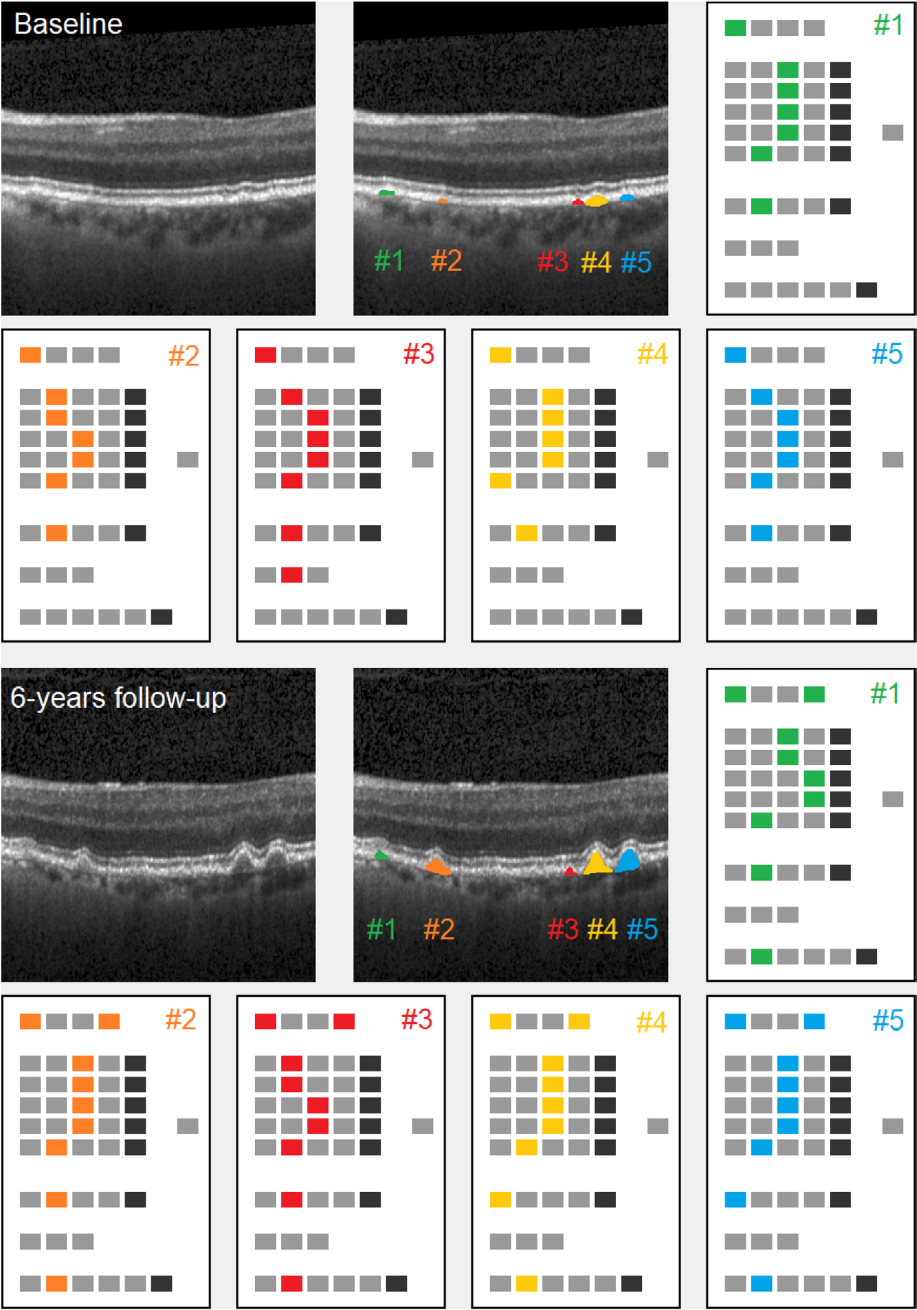
Lesion tracking (proband #27). First row, left: original baseline image (cutout), captured 21‐11‐2013. Middle: five lesions masked in different colours (cutout). Raw cutting areas are 2740, 1566, 2001, 7307 and 3262 μm^2^. First row, right, and second row: processed ‘EarlyAMDRate’ questionnaires for the five lesions, see Figure 1. Third row, left: original follow‐up image (cutout), captured 28‐11‐2019, the same position as at baseline (tracking function of OCT device enabled). Middle: five lesions masked (cutout), using the same colours as above. Raw cutting areas are 3219, 9917, 2740, 13 397 and 14 093 μm^2^. Third row, right, and fourth row: processed ‘EarlyAMDRate’ questionnaires, see Figure 1.

We provide a single example of lesion tracking from baseline to follow‐up, cf. Figure 9. The proband has been assessed twice at interval of about 6 years. At follow‐up, all five lesions identified at baseline showed noticeable growth. Observed sizes for lesions 1–5 increased by 478, 8351, 739, 6089 and 10 830 μm^2^, respectively. Cutouts of 300 × 300 pixels format are shown together with processed ‘EarlyAMDRate’ grading questionnaires.

### 3.4 Correlation between measurement units

The correlation between pixels and μm or μm^2^ measurement units has been assessed for *N* = 197 lesions, excluding the single lesion described as a hyper‐reflective focus. For the lesions analysed, the mean true pixel size is 11.27 × 3.87 μm^2^, and Pearson's correlation coefficient between the lesions' projected diameters measured in pixels and micrometres amounts to 0.9975 (*p* < 0.0005). The correlation coefficient between the cutting areas measured in pixels and square micrometres shows the same behaviour, amounting to 0.9991 (*p* < 0.0005). Consequently, pixels and μm or μm^2^ measurement units are almost perfectly positively correlated, and the subsequent analysis will be continued using metric units.

### 3.5 Distribution of raw lesion sizes

**FIGURE 10:**
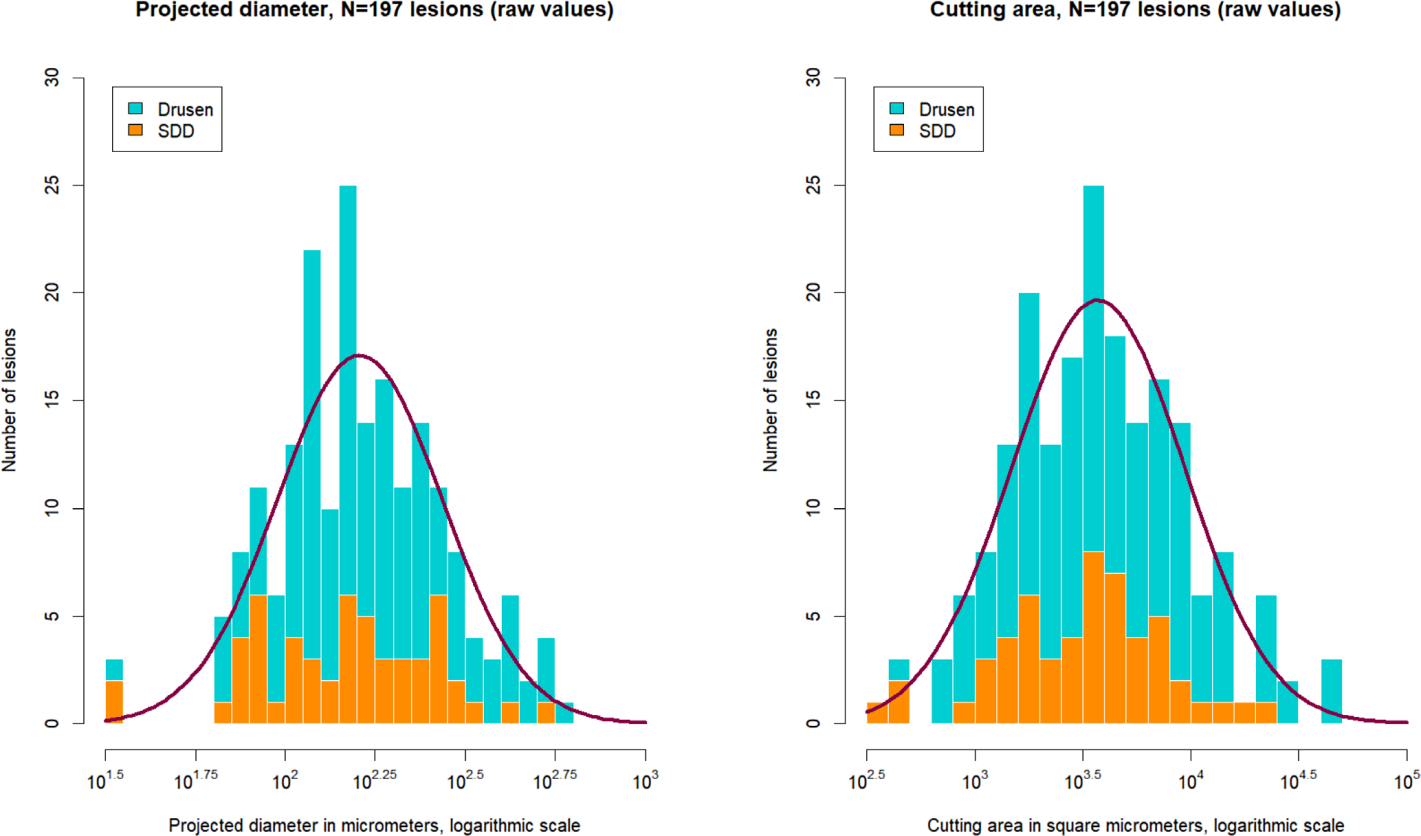
Distribution of projected diameter (left) and cutting area (right) for *N* = 197 lesions. Purple: fitting curve of log‐normal distribution. The single lesion consisting of hyper‐reflective material has been excluded.

Sample statistics were calculated for *N* = 197 lesions, excluding again the single lesion consisting of hyper‐reflective material only, see Table 2. As Figure 10 shows, the projected diameter as well as the cutting area follow log‐normal distributions within the sample. In both cases, Shapiro–Wilk tests to α = 0.05 level against null hypothesis of normal distribution for logarithmic values turned out insignificantly (with *p* = 0.0724 or *p* = 0.5409, respectively, Type II error levels not available). Note that observed lesion sizes are raw values without stereological corrections.

**TABLE 2:** Sample statistics for *N* = 197 lesions. The single lesion consisting of hyper‐reflective material only has been excluded from statistical analysis.

Entity	Minimum	Median	Mean ± St. dev.	Maximum
Projected diameter (pixels)	3	14	16.4 ± 9.3	51
Cutting area (pixels)	8	82	134.8 ± 165.2	1077
Projected diameter (μm)	32.5	154.8	185.1 ± 104.7	568.3
Cutting area (μm^2^)	336.4	3666.8	5904.1 ± 7192.2	46893.5

## 4 DISCUSSION

### 4.1 Detailed OCT‐based phenotyping of AMD lesions

In OCT scans, AMD lesions are captured in much more detail than in fundus photographs. Consequently, OCT‐based lesion grading should render possible (a) an unequivocal identification of lesions in their early and even earliest stages, (b) a detailed assessment of individual lesions and tracking of their development and interaction with the surrounding retinal features over time and (c) a general description of AMD stage and progression in terms of metric phenotypes (e.g., density of lesions, cumulative drusen volume or progression rates of the former) instead of categorical ones.

For these purposes, however, OCT‐based phenotyping of lesions already documented in the literature as yet is not suitable. Gattoussi et al. (2019), p. 367, classify lesions either as (isolated or confluent) drusen, SDDs or hyper‐reflective clumps, but ignore the interaction between lesions and surrounding layers as well as the lesion' brightnesses. Numerical measurements are confined to ‘the size of the largest subfoveal drusen and the location of the nearest drusen from the centre’ (ibid.). In another class of publications, an automated retina layer segmentation approach is pursued; see for example, Kananen and Immonen (2023), Lu et al. (2023), Section 2.3, and Schlanitz et al. (2017). The idea is that, in presence of drusen, the retinal volume enclosed between Bruch's membrane and the outer RPE layer is increased. This volume will be considered as a proxy for the cumulative drusen volume. Automated layer segmentation, however, is prone to a number of methodological problems, cf. Wagner et al. (2025b), Section 4.3. Layer segmentations and volume measurements are mostly based on undocumented, ‘black‐box’ commercial software packages provided by the OCT device manufacturers, the quality of segmentations is often very poor, cf. Brandl et al. (2019), and results are strongly device‐dependent. Even if these restrictions are accepted, this approach can provide estimates of cumulative lesion volume only but no description of individual lesions that is adequate to the precision of the underlying OCT imaging.

With the grading instrument ‘EarlyAMDRate’ presented here, we provide a flexible and pragmatic approach, which is oriented to the description and measurement of individual lesions. The basic categories for lesion description from Gattoussi et al. (2019) have been complemented by a detailed assessment of the immediate surroundings of the lesion as well as a description of the lesion's brightness and state of progression. As the examples in Section 3 show, by application of ‘EarlyAMDRate’ grading, objectives (a) and (b) stated above can be reached. In most cases, the basic distiction between conventional drusen and SDDs can be carried out by assessing the state of the outer RPE layer and Bruch's membrane around the lesion. For example, the lesion marked in Figure 5 clearly affects (and even disrupts) the outer RPE layer, thus being classified as a drusen, while the lesion in Figure 6 is situated above the outer RPE layer and leaves it unaffected, thus being classified as a SDD. Objective (c) can be achieved by the ‘EarlyAMDRate’ instrument as well. Measurement of lesion size from masking enables the generation and evaluation of metric, lesion‐oriented phenotypes for AMD description. Numbers of lesions as well as individual and cumulative lesion sizes can be obtained and differentiated by lesion types and actually caused damages. To the best of the authors' knowledge, information about log‐normal distribution of lesion sizes is documented first here, cf. Figure 10.

The ‘EarlyAMDRate’ questionnaire reflects adequately the detailed view of a lesion enabled by OCT imaging but avoids to get lost in overly precise definitions. For example, we refrain from a possible determination of numeric thresholds for the brightness categories ‘low’, ‘moderate’ and ‘high’, believing that such definitions are premature at the present time.

### 4.2 Limitations

Of course, the approach presented here has several limitations. First of all, in terms of time and staff, the manual grading step is still highly expensive. Furthermore, there is no evaluation of intra‐ or intergrader variability as yet. Note that, even within an OCT image, the assessment of layer affection/damage around a lesion is not unambiguously possible in all cases. For example, for the lesion in Figure 7, the state of Bruch's membrane cannot be clearly assessed.

Two further methodological limitations are inherent to the process of OCT image generation itself. First, due to the undersampling in most OCT acquisition protocols, resulting in a typical distance of 50–60 μm between adjacent B‐scans, lesions of small diameter may still be overlooked. Second, a stereological correction of all obtained lesion sizes is unavoidable; see a forthcoming paper of the authors.

## 5 CONCLUSION

The ‘EarlyAMDRate’ grading instrument for Early‐AMD lesions in OCT scans allows for unprecedented detail of description for single lesions, which is adequate to the precision of the underlying OCT imaging. The instrument enables an unequivocal identification of lesions in its early and even earliest stages, and a reliable differentiation of different lesion types, such as drusen and SDDs. Moreover, obtained grading information allows for tracking of lesions and their properties over time, as well as for generation of well‐differentiated metric phenotypes for the description of Early‐AMD. Application to a consecutive sample of *N* = 100 Early‐AMD patients uncovers a log‐normal distribution of observed lesion diameters and cutting sizes.

## AUTHOR CONTRIBUTIONS

Marcus Wagner: Conceptualization (equal); methodology (equal); visualization (lead); writing – original draft (lead); writing – review and editing (equal). Thomas Peschel: Conceptualization (equal); writing – review and editing (equal). Carla J. Leutloff: Conceptualization (equal); data curation (equal); investigation (equal); methodology (equal); writing – review and editing (equal). Franziska G. Rauscher: Conceptualization (equal); data curation (equal); investigation (equal); methodology (equal); resources (lead); writing – original draft (supporting); writing – review and editing (equal). All authors read and approved the final manuscript.

## FUNDING INFORMATION

FGR and MW were supported by the German Research Foundation (grant no. 497989466). Research with LIFE data was supported by the Leipzig Research Center for Civilization Diseases (LIFE), Leipzig University, which is funded by the EU, the European Social Fund, the European Regional Development Fund and Free State Saxony's Excellence Initiative (project numbers 713‐241202, 14505/2470 and 14575/2470).

## CONFLICT OF INTEREST STATEMENT

All authors declare no conflicts of interest.

## ETHICS STATEMENT

The LIFE study follows the tenets of the Declaration of Helsinki and was approved by the responsible institutional ethics board of the Medical Faculty of Leipzig University (approval numbers 2632009‐14122009, 263/09‐ff, 201/17‐ek). Written informed consent was obtained from all participants. For the present research, data from LIFE‐Adult Baseline and 6‐years follow‐up were used. Data use was approved by the institutional review board of the Leipzig Research Center for Civilization Diseases (LIFE).

## APPENDIX A Working instructions for the application of the ‘EarlyAMDRate’ instrument

Paragraphs marked with an asterisk * only apply if the B‐scan under consideration is a follow‐up image, which is assessed together with its related baseline scan.

### A.1. General properties of the B‐scan

#### A.1.1. Identifier

Keep the filename/identifier of the B‐scan under consideration, the number of the lesion assessed within, and the colour used for masking it. * If necessary, record the filename/identifier of the related baseline scan as well.

#### A.1.2. Global B‐scan properties (fields GEN‐01 to GEN‐04)

If necessary, tick more than one field.

GEN‐01: Tick the field if the B‐scan is gradable at all.

GEN‐02: Tick the field if signs of dry Late‐AMD are visible in the B‐scan.

GEN‐03: Tick the field if signs of wet Late‐AMD are visible in the B‐scan.

GEN‐04: Tick the field if the B‐scan under consideration is a follow‐up image that is assessed together with its related baseline scan.

### A.2. Masking of a single AMD lesion

#### A.2.1. Rules for masking

Within a single B‐scan, every AMD lesion should be masked with a different colour. Do not use white, black or greyscale tones. Every lesion mask must form a connected area (horizontal, vertical or diagonal connections between pixels are possible). The minimal size of a mask is 2 pixels.

#### A.2.2. *Treatment of lesions in pairs of related baseline and follow‐up scans

If a pair of related baseline and follow‐up scans is available for grading, then consider the following instructions.

1. First, identify the pairs of unambiguously related single lesions in both scans. For these, select the same colours for masking in both grading processes.
2. If two or more lesions from baseline merge into a single confluent lesion at follow‐up, then divide the latter into appropriate subareas bordered by verticals at the notches' positions (thus counting the parts of the confluent follow‐up lesion as separate entities).
3. If a lesion from baseline completely disappeared at follow‐up, then mark its former location with a single pixel of the same colour as in baseline. Note that, by the rule in A.2.1., this single pixel is unambiguously distinguished from a mask of a present lesion, which must consist of at least two connected pixels.
4. If a lesion appears newly at follow‐up, then select a colour not appearing in the baseline grading.
5. If a lesion in follow‐up is not newly formed but cannot be unambiguously related to a corresponding lesion in the baseline scan, then select a colour not appearing in the baseline grading as well.

### A.3. Processing the questionnaire

#### A.3.1. Assessment of lesion position and surrounding layer structure (five rows with fields LAY‐01A to LAY‐05E and INSEP)

Into the assessment, the following five layers are included: external limiting membrane (LAY‐01 with fields LAY‐01A to LAY‐01E), ellipsoid zone (LAY‐02 with fields LAY‐02A to LAY‐02E), interdigitation zone (LAY‐03 with fields LAY‐03A to LAY‐03E), outermost RPE layer (LAY‐04 with fields LAY‐04A to LAY‐04E) and Bruch's membrane (LAY‐05 with fields LAY‐05A to LAY‐05E)

If a layer LAY‐xx is clearly identifiable around the lesion, then tick either

LAY‐xxA: if the layer is bent or bulged downwards but still connected, or

LAY‐xxB: if the layer is unchanged and intact, or

LAY‐xxC: if the layer is bent or bulged upwards but still connected, or

LAY‐xxD: if the layer is disrupted or destroyed.

Otherwise, tick LAY‐xxE if the state and geometry of the layer cannot be clearly assessed or are not gradable at all.

Tick precisely one field per row.

INSEP: Tick this box if the interdigitation zone and outer RPE layer cannot be clearly separated around the lesion. In this case, set the ticks in both the rows LAY‐03 and LAY‐04 at the same position.

#### A.3.2. Brightness of the lesion (fields BRI‐01 to BRI‐05)

Tick precisely one of the following fields. Masked lesion area has either

BRI‐01: low brightness, or
BRI‐02: moderate brightness, or
BRI‐03: high brightness, or
BRI‐04: shows other brightness properties or patterns (e.g. strongly varying brightness throughout the lesion's cut interior), or
BRI‐05: its brightness properties cannot be clearly assessed or are not gradable at all.

#### A.3.3. Special properties of the lesion (fields SP‐01 to SP‐03)

If necessary, you may tick more than one box.

SP‐01: Tick the field if the lesion is confluent with another adjacent lesion.
SP‐02: Tick the field if, besides of the lesion itself, trailing hyper‐reflective material (‘drusen ooze’) is visible in the B‐scan which is clearly related to this lesion.
SP‐03: Tick the box if the lesion itself consists of hyper‐reflective material only, which is related to visible layer deformation or destruction.

#### A.3.4. *Assessment of progression (fields PRO‐01 to PRO‐06)

If field GEN‐04 is not ticked, that is, the B‐scan under consideration is assessed for itself (and not compared with a predecessor scan from baseline) then tick no fields in this row. Otherwise, assess the state of progression for the lesion under consideration and tick precisely one of the following fields.

PRO‐01: if, compared with baseline, the lesion is newly formed, or
PRO‐02: if, compared with baseline, the lesion shows noticeable growth, or
PRO‐03: if, compared with baseline, the lesion is stationary, or
PRO‐04: if, compared with baseline, a partial or complete remission of the lesion occurred and the layer structure around the lesion is preserved, or
PRO‐05: if, compared with baseline, a partial or complete remission of the lesion occurred but the layer structure around the former position of the lesion is destroyed, or
PRO‐06: if the progression status of the lesion cannot be clearly assessed or is not gradable at all. For example, this case may occur if a given lesion in follow‐up is not newly formed but cannot be unambiguously related to a corresponding lesion in the baseline scan.

#### A.3.5. *Special instructions

If a pair of related baseline and follow‐up scans is simultaneously graded, then further consider the following special instructions.

1. If two or more lesions from baseline merge into a single confluent lesion at follow‐up, then consider the parts of the confluent follow‐up lesion as separate entities not only in the masking but in the processing of questionnaires as well. Consequently, assess for every part its position and affection of layers, brightness, special properties and progression as if it were a separate lesion. Of course, tick SP‐01 for all parts.
2. If a lesion from baseline completely disappeared at follow‐up, then process the questionnaire for this lesion at follow‐up by ticking LAY‐01E, LAY‐02E, LAY‐03E, LAY‐04E, LAY‐05E and BRI‐05. Moreover, tick either PRO‐04 or PRO‐05.
